# Stiffness Tuning and Ductility Enhancement of Semicrystalline Polymers by Incorporating Miscible Noncrystallizable Blocks

**DOI:** 10.1021/acsmacrolett.6c00295

**Published:** 2026-07-23

**Authors:** Katherine M. Gunter, Richard A. Register

**Affiliations:** Department of Chemical and Biological Engineering, 6740 Princeton University, Princeton, New Jersey 08544-5263, United States

## Abstract

The mechanical behavior of hydrogenated poly­(norbornene-*b*-substituted norbornene) diblock copolymers was investigated, where the substituted norbornene blocks are intimately mixed within the amorphous layer between hydrogenated polynorbornene (N) crystallites. In particular, hydrogenated poly­(norbornene-*b*-norbornyl norbornene) diblock copolymers were studied; hydrogenated poly­(norbornyl norbornene) homopolymers are glassy at room temperature. Enhancement of both yield stress and Young’s modulus was found at an enrichment of just 20 wt % glassy (G) block in the amorphous layer. Surprisingly, these N-G diblocks exhibit improved toughness over N homopolymers, with a nearly 4× decrease in minimum tie molecule fraction for ductility compared to quenched N homopolymers. Enhanced ductility is observed whether the substituted norbornene block is glassy or rubbery, as was demonstrated with a hydrogenated poly­(norbornene-*b*-hexylnorbornene) diblock copolymer (N-R). These materials’ strain-hardening moduli reveal that the amorphous block promotes ductility by reducing disentanglement of tie molecules during drawing.

## Graphical abstract

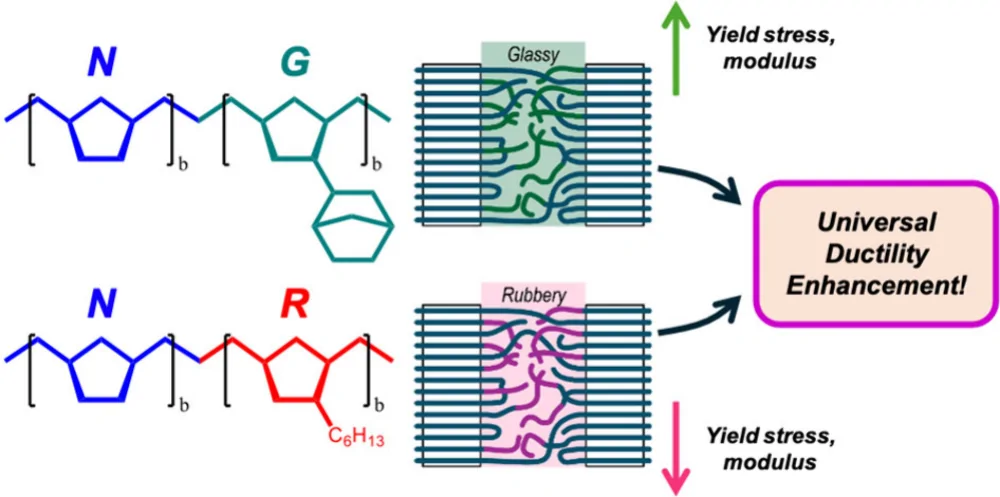

Blending a semicrystalline polymer with a rubber can yield a composite material with enhanced toughness and ductility. In practice, polypropylene^1–3^ and nylon-66 ^4,5^ benefit greatly from rubber toughening. As described by Wu^2^ and later elaborated upon by Corté and Leibler,^6^ the average distance between rubber domains (ligament thickness) must be reduced below a critical value to achieve toughening. However, simple melt-mixing of two polymers can prove insufficient to achieve the small domain sizes necessary for toughening. To obtain these smaller and more stable domain sizes, a block or graft copolymer of the two components may be added, or the entire rubber fraction may be incorporated as a block or graft.^7^ However, microphase separation in such block copolymers can complicate processing by raising melt viscosity and elasticity.^8,9^

An alternative means of property modification is to incorporate noncrystallizable blocks (either rubbery or glassy) into the polymer chain that are too short to induce microphase separation. In these cases, no distinct domains of the amorphous block are present. All constituent blocks are homogeneously mixed in the melt; in the semicrystalline solid, the noncrystallizable blocks are intimately mixed with the amorphous fraction of the crystallizable block, between adjacent crystallites within a lamellar stack. These noncrystallizable blocks can either stiffen or soften the amorphous layer, depending on the glass transition temperature (*T*_g_) of their corresponding homopolymer. Using this approach, our group has previously explored the properties of polyethylene-hydrogenated poly­(norbornyl norbornene) diblock copolymers (PE-hPNbN).^10^ The high-*T*_g_ hPNbN block (*T*_g_ = 115 °C)^11^ was found to increase both the yield stress and Young’s modulus, though not without limitations: there is a maximum total molecular weight to avoid microphase separation in the melt, dictated by the strength of the repulsive interactions between the PE-hPNbN pair (Flory interaction parameter χ, or interaction energy density *X*).^11^ At these restricted molecular weights, many of these PE-hPNbN diblock copolymers possessed low tie molecule concentrations and were therefore brittle, making it difficult to ascertain how the high-*T*_g_ block impacted bulk toughness along with other high-strain mechanical properties.

We build upon this previous work by elucidating how mechanical behavior at both low and high strains can be manipulated by the incorporation of short, melt-miscible amorphous blocks. Using our previous study into the brittle-ductile transition of hydrogenated polynorbornene (hPN) homopolymers and random copolymers as a foundation,^12^ we can synthesize hPN-hPNbN diblock copolymers with an expectation of whether the hPN block alone would be brittle or ductile. hPN also offers two distinct advantages over PE, the first of which is that the hPN-hPNbN pair has a much lower *X* compared to PE-hPNbN.^11,13^ This allows for the synthesis of higher molecular weight (and therefore more ductile) materials while avoiding microphase separation in the melt. Second, the *T*_g_ of amorphous hPN (4 °C^14^) is much higher than that for PE.^15,16^ Less hPNbN is therefore required to stiffen the amorphous layer. Also, because *T*_g_ of hPN is close to room temperature, and dynamic mechanical analysis of hPN homopolymers reported by Klein et al. revealed that hPN had yet to fully enter its rubbery plateau at room temperature,^17^ it is plausible that the inclusion of a short rubbery block will soften the amorphous layer. Hydrogenated polyhexylnorbornene (hPHxN) is used here as a rubbery block due to its low *T*_g_ (−22 °C).^14^ Diblock copolymers were synthesized using living ring-opening metathesis polymerization (ROMP) initiated with a Schrock-type initiator to provide targeted molecular weights and low dispersities.^18^ All polymers were then hydrogenated with a homogeneous ruthenium-based catalyst which does not induce epimerization of the hPN cyclopentylene backbone ring.^19^ Details of the synthesis are provided in the Supporting Information.

A total of four diblock copolymers were examined: three containing a short hPNbN end block (N-G, with “G” denoting “glassy”) and one containing a short hPHxN end block (N-R, with “R” denoting “rubbery”). All four contain roughly identical weight fractions of the short amorphous hydrogenated poly­(alkylnorbornene) block (*w*_hPaN_ ∼ 0.11), as confirmed with ^1^H NMR spectroscopy (see Supporting Information, Figures S1 and S2); the three N-G diblocks vary systematically in number-average molecular weight (*M*_*n*_). Polymers are coded by the number-average molecular weights (in kg/mol) of their respective blocks, e.g., “34N-4G” consists of a 34 kg/mol N (hPN) block and a 4 kg/mol G (hPNbN) block, while “41N” is a 41 kg/mol N (hPN) homopolymer. Quenched (Q, cooled from the melt at ∼1000 °C/min) and slow-cooled (SC, cooled at ∼1 °C/min) films of each polymer were prepared, allowing for direct comparison between diblocks and N homopolymers with analogous thermal histories, which we analyzed previously.^12^

The characteristics of these polymers and their crystallized films are detailed in Table 1. All polymers exhibit narrow molecular weight distributions (*Đ* < 1.15), and high crystallinities at room temperature. The short amorphous block appears to have a negligible impact on N block crystallinity; note that the overall values of weight fraction crystallinity (*w*_c_) reported in Table 1 are not normalized for the ∼11 wt % of the material contained in the amorphous block.

**Table 1:** Characteristics of Diblock Copolymers Synthesized for This Work, And Their Molded Films, Compared with N Homopolymers Analyzed Previously^12^

thermal history	polymer	*M*_n_ ^a^ (kg/mol)	*Đ*	*w*_hPaN_ ^b^	ε_b_ ^c^ (%)	σ_*y*_ ^c^ (MPa)	*E* ^c^ (MPa)	⟨*G*_p_⟩^c^ ^,^ ^d^ (MPa)	*w*_c_ ^e^	*T*_g,am_ ^f^ (°C)	*d* (nm)	*L*_c_ (nm)	log_10_(*P*_avg_)^g^
Q	34N-4G^h^	38.3	1.09	0.117	160 ± 92	20.7 ± 0.6	1030 ± 55	^h^	0.54	26	26.4	13.9	–2.70
	50N-7G	57.2	1.07	0.121	690 ± 19	14.9 ± 0.4	720 ± 53	53 ± 2	0.50	25	26.4	12.9	–1.93
	77N-10G	86.6	1.12	0.110	490 ± 21	13.6 ± 0.2	600 ± 39	87 ± 4	0.46	21	29.3	13.2	–1.54
	80N-10R	90.0	1.13	0.112	660 ± 25	10.2 ± 0.2	230 ± 14	100 ± 3	0.46	–2	30.0	13.2	–1.53
	41N^h^	41.1	1.07	0	27 ± 6	16.8 ± 0.4	720 ± 55	^h^	0.52	4^i^	25.2	12.7	–2.14
	74N	73.8	1.07	0	820 ± 34	13.0 ± 0.2	450 ± 12	27 ± 0.4	0.53	4^i^	28.7	14.7	–1.66
SC	34N-4G	38.3	1.09	0.117	^j^	^j^	^j^	^j^	0.69	37	35.8	24.2	–4.75
	50N-7G	57.2	1.07	0.121	1.7 ± 1.1	^k^	1800 ± 36	^k^	0.62	32	35.6	21.8	–3.48
	77N-10G^h^	86.6	1.12	0.110	91 ± 35	31.5 ± 0.3	1640 ± 34	^h^	0.62	29	40.1	24.2	–2.90
	80N-10R	90.0	1.13	0.112	570 ± 8	16.5 ± 0.03	420 ± 27	25 ± 0.1	0.63	–5	41.2	25.3	–2.93
	41N	41.1	1.07	0	1.5 ± 0.5	^k^	810 ± 99	^k^	0.72	4^i^	35.6	25.0	–4.40
	74N^h^	73.8	1.07	0	38 ± 4	20.3 ± 0.2	760 ± 32	^h^	0.65	4^i^	37.0	23.5	–2.80

a Number-average molecular weight of saturated polymer, adjusted from the unsaturated value.

b Weight fraction of the amorphous block in the saturated polymer.

c Average ± one standard deviation over three replicates.

d Strain-hardening modulus, ⟨*G*_p_⟩, see Supporting Information for calculation.

e Calculated by dividing the melting enthalpy by 96 J/g, the melting enthalpy of the perfect hPN crystal.^12^

f Predicted *T*_g_ of mixed amorphous fraction, calculated from the Fox equation.

g Tie molecule fraction calculated with *L*_crit_ set equal to *d* + *L*_c_, see Supporting Information.

h Specimen failed in transition mode (*P*_avg_ ≈ *P*_BDT_) and did not exhibit strain-hardening.

i *T*_g_ of amorphous hPN.^14^

j Material too brittle for mechanical testing.

k Specimen failed prior to yielding.

To confirm that these diblocks are “melt-miscible” (where dissimilar blocks are intimately mixed in the melt, alternatively termed “disordered” or “homogeneous”), high-temperature small-angle X-ray scattering (SAXS) was performed on 77N-10G and 80N-10R. Specimens were heated *in situ* to 160 °C and cooled through crystallization while taking SAXS patterns at 5 or 10 °C intervals. Figure 1 shows these SAXS patterns, where both materials exhibit featureless melts at 160 °C, showing significant scattering only after crystallization. Sufficient electron density contrast exists between the molten N and G blocks, as well as the N and R blocks, for microphase-separation to be detected, were it present (see Supporting Information). In 80N-10R (Figure 1b), the scattering present at 135 °C results from isothermal crystallization (each SAXS pattern was acquired for 15 min). Since 50N-7G and 34N-4G possess lower *M*_n_ values than 77N-10G, and the hPN-hPNbN pair exhibits upper-critical ordering transition behavior,^13^ these diblocks will also possess homogeneous melts.

**Figure 1:**
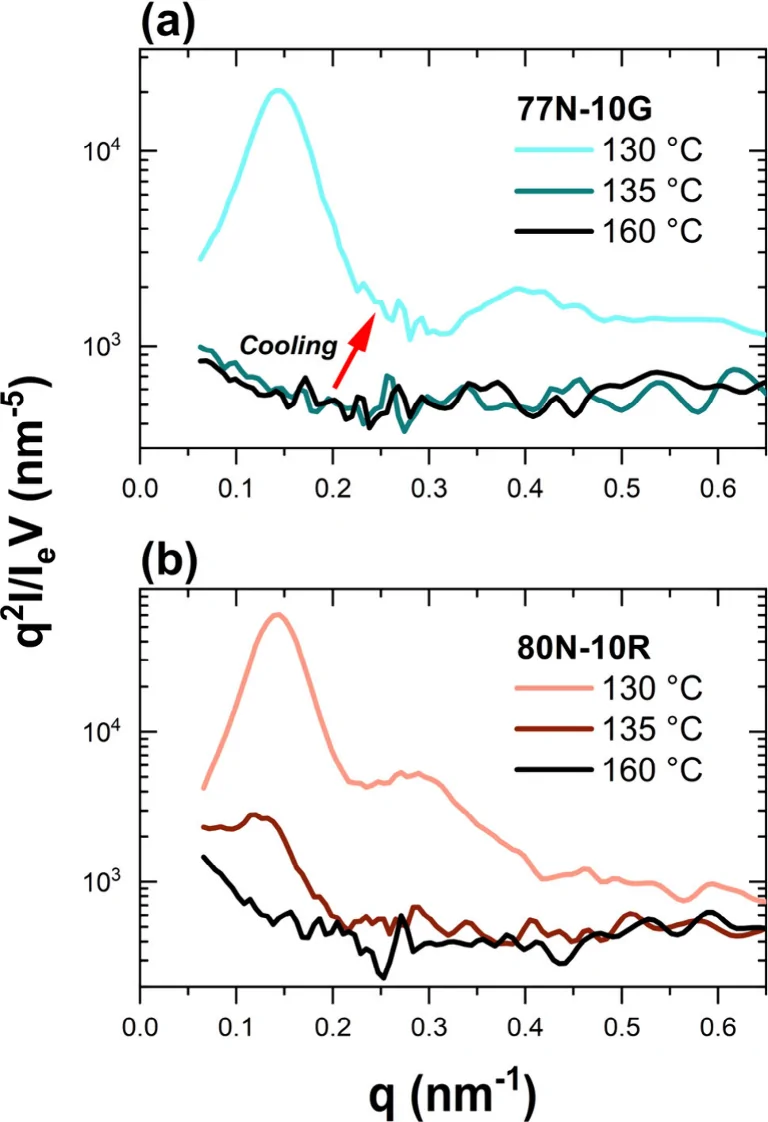
High-temperature SAXS patterns of (a) 77N-10G and (b) 80N-10R cooled *in situ* from the melt.

Stress–strain curves for all quenched and slow-cooled N-G films are shown in Figure 2, where the stress–strain curve for the 74N homopolymer, quenched or slow-cooled from the melt, is also included for direct comparison. The breaking strain (ε_b_), yield stress (σ_*y*_), and Young’s modulus (*E*) are all listed in Table 1. Focusing first on the low-strain properties, the G block clearly raises σ_*y*_ and *E* relative to the hPN homopolymer, though this effect is diminished when the material is quenched. Additionally, as *M*_n_ increases across the series, σ_*y*_ for the quenched N-G diblocks decreases, approaching that of the quenched N homopolymer. *E* in quenched N-G diblocks also drops with increasing *M*_n_ across the series (from 1030 to 600 MPa), though it does not decline to the same value as that for the quenched homopolymer (450 MPa for 74N Q). Nevertheless, the drops in *E* and especially in σ_*y*_ exceed those anticipated for the modest changes measured in *w*_c_ and crystal thickness *L*_c_, which are the generally accepted parameters controlling *E* and σ_*y*_, respectively.^20–23^

**Figure 2:**
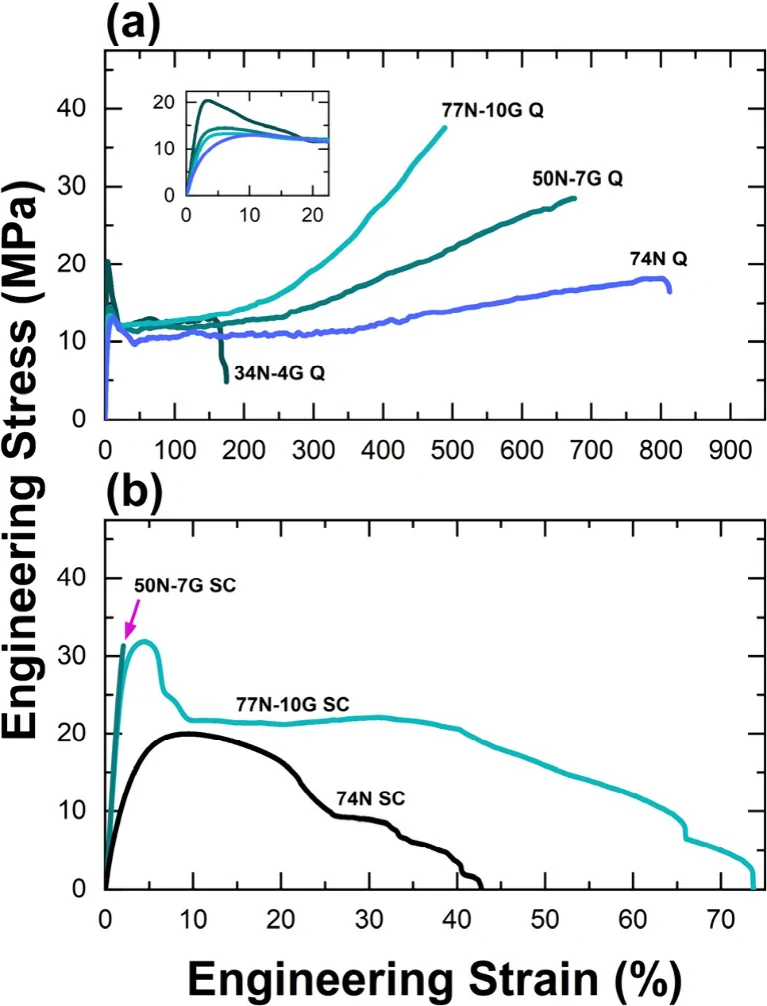
(a, b) Stress–strain curves for one representative specimen of each N-G diblock (a) quenched and (b) slow-cooled from the melt, compared against 74N. Inset in (a) shows the low-strain region.

Instead, the observed trends in *E* and σ_*y*_ can be traced to variations in the fraction of the high-*T*_g_ block within the amorphous layer, and how those variations can drive the *T*_g_ of the mixed amorphous layer up toward room temperature, thereby increasing *E* and σ_*y*_. The average weight fraction of hPNbN in the amorphous phase, *w*_hPNbN,am_, is easily calculated with eq 1:

1 $$w_{\mathrm{hPNbN,am}}=\frac{w_{\mathrm{hPNbN}}}{1-w_{c}}$$

Figure 3 shows the relationship between the two low-strain properties and *w*_hPNbN,am_, where data for both the quenched and slow-cooled N-G diblocks appear to collapse onto the same trends. Ultimately, this reveals two facts about this system: the first is that *w*_hPNbN,am_ is a good predictor of the low-strain properties, while the second is that the low-strain mechanical properties are not significantly different from those of the homopolymer when *w*_hPNbN,am_ is lowered to 20 wt %. For the PE-hPNbN diblock copolymers our group has previously examined,^10^ the minimum *w*_hPNbN,am_ for any significant increase to either σ_*y*_ or *E* was 40%, double what we find here for N-G diblocks. This phenomenon stems from the much higher *T*_g_ of hPN relative to PE, such that the mixed amorphous layer in N-G diblocks has a *T*_g_ approaching room temperature. The Fox equation, written here for a multicomponent system in eq 2, may be used to estimate the amorphous layer *T*_g_ (*T*_g,am_):

2 $$\frac{1}{T_{g}}=\overset{n}{\underset{i}{\sum }}\frac{w_{i}}{T_{g,i}}$$

where *n* is the number of components. Estimated *T*_g,am_ values for all specimens are listed in Table 1. For 77N-10G Q, *T*_g,am_ is 21 °C, just below room temperature. While this is the average value, we anticipate there to be a compositional gradient across the amorphous layer, with the G block enriched at the crystal fold surface.^10,24^ It is therefore very likely that the *T*_g_ at the N crystal fold surface is above room temperature, hindering the fine-slip mechanism that occurs at yielding.^25,26^

**Figure 3:**
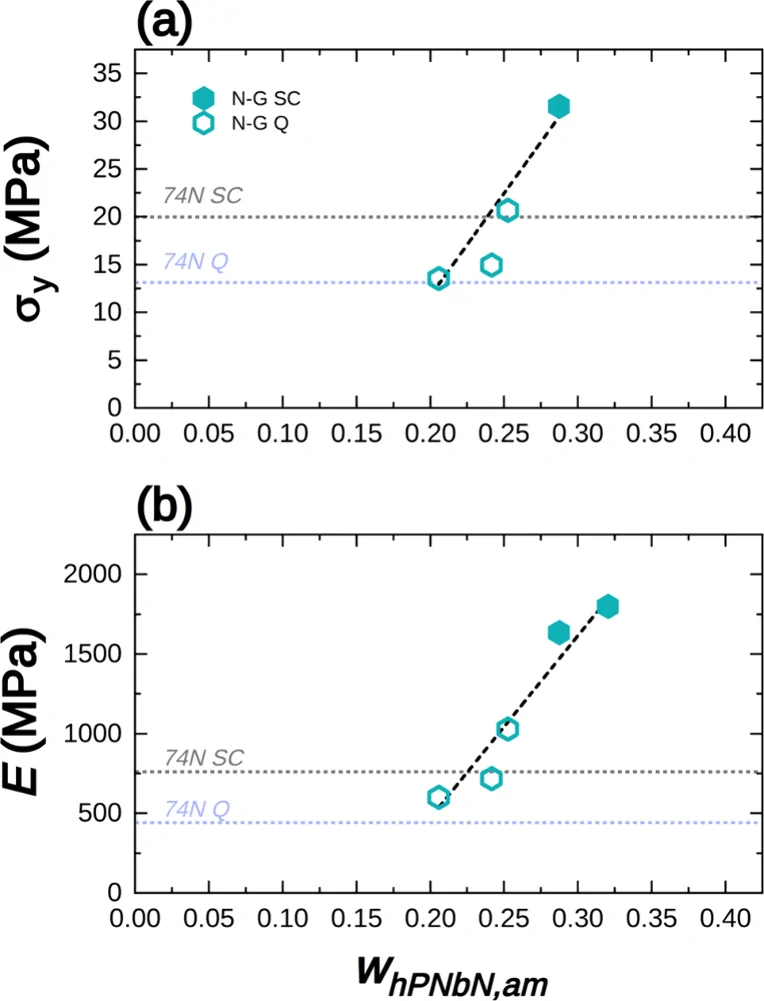
(a) Yield stress and (b) Young’s modulus plotted against the average weight fraction of hPNbN in the amorphous phase, *w*_hPNbN,am_. Dotted horizontal lines indicate the corresponding values for the 74N homopolymer. Dashed lines through the data are guides for the eye.

Next examining ductility, we see that for each thermal history (Q and SC), one polymer (34N-4G Q and 77N-10G SC) was observed to yield, but the resulting neck was unstable and failed to propagate the full gauge length. These materials thus failed in a “transition mode” between brittle and fully ductile failure.^12,27^ A material failing in transition mode possesses enough tie molecules to resist brittle fracture, but an insufficient quantity for the fibrils formed postyield to be stable under an applied tensile strain. Our group has previously applied the Huang-Brown model^28,29^ to calculate the tie molecule fraction at the brittle-ductile transition for PE^27^ and hPN^12^ homopolymers and random copolymers of varying molecular weights; using an identical approach here (see Supporting Information for equations and additional considerations), tie molecule fractions (*P*_avg_) were calculated for all materials. These values are listed in Table 1. Materials failing in transition mode are, by definition, close to the brittle-ductile transition (BDT), so their tie molecule fractions (*P*_avg_) closely correspond to those at the BDT (*P*_BDT_). A comparison of the *P*_avg_ values for the two quenched polymers failing in transition mode (34N-4G Q and 41N Q), which have *P*_avg_ ≈ *P*_BDT_, shows that *P*_BDT_ for quenched N-G diblocks is substantially lower (∼4×) than *P*_BDT_ for quenched N homopolymers. In other words, these crystalline-glassy diblock copolymers require a factor of 4 fewer tie molecules to resist brittle fracture than N homopolymers. For the slow-cooled films, this difference is considerably smaller (∼1.3×, comparing 77N-10G SC to 74N SC), but the fact that there is a difference at all is interesting, as it implies that these glassy blocks improve ductility. Our initial intuition would be that hPNbN (G block) would embrittle the bulk material by making the amorphous layer glassy, as has been observed in high-*T*_g_ semicrystalline polymers such as poly­(lactide).^30–32^ Following our previous results for hPN homopolymers and random copolymers,^12^ this reduction in *P*_BDT_ indicates that the incorporation of a short G block reduces the fraction of tie molecules which are lost during the lamellar fragmentation which accompanies drawing. It should also be emphasized that in N homopolymers and random copolymers, we found a strong correlation between the fraction of tie molecules lost and the initial crystal thickness, *L*_c_. For a given thermal history, the N-G diblocks have nearly identical values of *L*_c_ as the N homopolymers (Table 1), indicating that it is the amorphous block, and not an increase in crystal thickness, that is inhibiting tie molecule disentanglement.

To study this further, we analyze the mechanical behavior of the N-R diblock copolymer, 80N-10R. If tie molecule loss during an applied strain were dependent on amorphous layer segmental dynamics, N-R and N-G diblocks would be anticipated to qualitatively differ in their behavior. Figure 4 shows the stress–strain curves of quenched and slow-cooled 80N-10R in relation to 77N-10G and 74N, demonstrating that 80N-10R also features enhanced ductility in relation to the corresponding N homopolymer. This is most evident when these materials are slow-cooled (Figure 4b), where both 77N-10G and 74N fail in transition mode, while 80N-10R is fully ductile. This ductility improvement occurs in tandem with a 25% decrease in tie molecule fraction relative to 74N (Table 1), indicating that the R block, like the G block, promotes ductility despite the opposite effects that R vs. G blocks have on *T*_g,am_. The low-strain mechanical properties, by contrast, match our expectations based on *T*_g_: quenched and slow-cooled 80N-10R exhibits substantially lower σ_*y*_ and *E* than do N homopolymers processed analogously, qualitatively opposite from the behavior of N-G diblocks.

**Figure 4:**
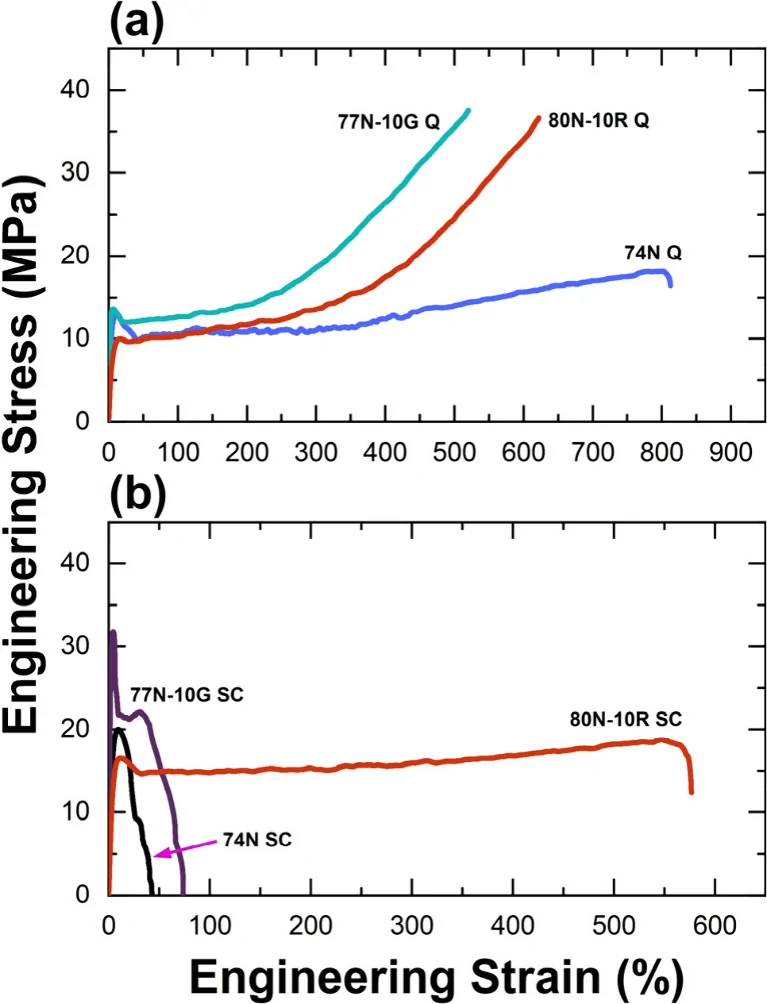
Stress–strain curves for one representative specimen of each of three polymers containing N blocks of similar length (∼77 kg/mol: 77N-10G, 80N-10R, and 74N), either (a) quenched or (b) slow-cooled.

To confirm that reduced tie molecule loss during lamellar fragmentation is the source of improved ductility and reduced *P*_BDT_, the strain-hardening modulus (⟨*G*_p_⟩) of each material was determined. Kurelec et al. showed that ⟨*G*_p_⟩ is a strong predictor of a material’s environmental stress crack resistance and therefore reflects tie molecule content.^33^ Kida and associates later determined via Raman spectroscopy that strain-hardening behavior is governed by tie chains in drawn polymeric fibrils.^34–36^ Figure 5 shows ⟨*G*_p_⟩ (see Supporting Information for calculation from the stress–strain data), plotted against *P*_avg_ for all quenched materials, including several N homopolymers which we have previously reported.^12^ Here, ⟨*G*_p_⟩ for quenched specimens of both N-G and N-R diblocks appear to collapse onto the same trend, and are crucially shifted to the left of (or, equivalently, above) the quenched N homopolymer data. In other words, these diblock copolymers exhibit a significantly larger ⟨*G*_p_⟩ than an N homopolymer with the same tie molecule content in the undrawn specimens (*P*_avg_). Therefore, the data in Figure 5 reveal that these diblock copolymers experience reduced tie chain disentanglement during drawing.

**Figure 5:**
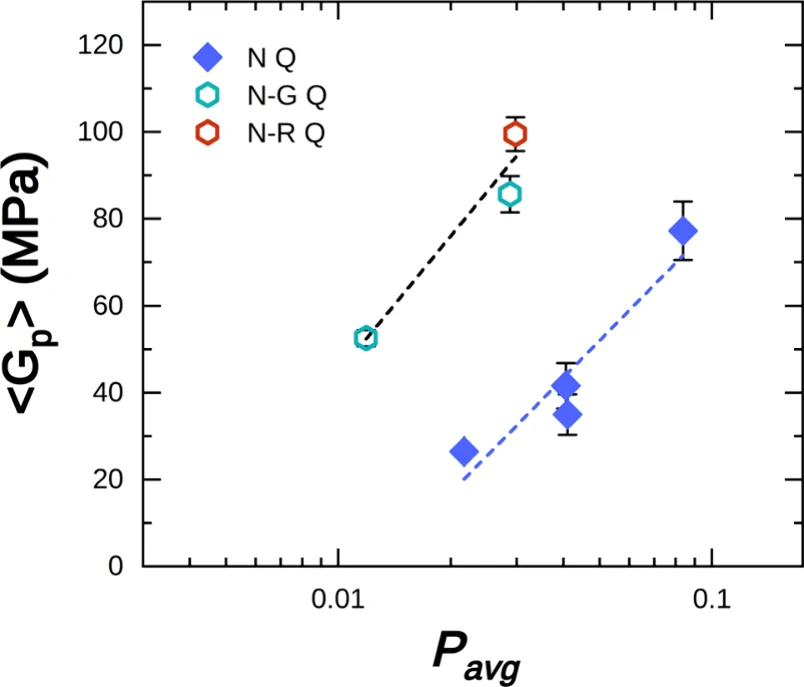
⟨*G*_p_⟩ for quenched N homopolymers, and N-G and N-R diblocks, plotted against *P*_avg_.

Due to their position at the end of the chain, the amorphous blocks cannot transmit applied stresses, so such an increase in ductility and reduction in *P*_BDT_ is unexpected. Though melt-miscible, the amorphous blocks still exhibit repulsive interactions with the N segments, disfavoring block mixing. Extensive chain reorganization necessarily accompanies lamellar fragmentation in all semicrystalline polymers.^37–40^ But in the present diblocks, the N crystal stems, which consist entirely of N segments, will experience an increased enthalpic penalty when being “pulled through” a mixed (N-G or N-R) amorphous layer, as compared with the pure-N amorphous layer in N homopolymers. We speculate that this additional penalty could reduce tie molecule loss during drawing, thereby enhancing ductility, regardless of whether the amorphous block is glassy or rubbery. These results reveal that ductility enhancement can be achieved via a route which is complementary to conventional rubber toughening, further highlighting the critical role of the amorphous layer in the deformation mechanism of semicrystalline polymers.

## Supplementary Material



## Notes

Materials; Polymer synthesis methods; Molecular weight distributions; ^1^H NMR spectra; Hydrogenation methods; Room-temperature SAXS patterns; High-temperature SAXS calibration curve; DSC first-heat melting endotherms and corresponding thermal properties; Methods for mechanical analysis; High-temperature SAXS and mechanical properties of higher-*M*_n_ diblocks exhibiting microphase-separated melts; Huang-Brown model equations for calculating tie molecule content (PDF)
