# Supplementary material for: Stiffness Tuning and Ductility Enhancement of Semicrystalline Polymers by Incorporating Miscible Noncrystallizable Blocks
Source: ACS Macro Lett. 2026 Jul 23;15(8):1129–34. doi: 10.1021/acsmacrolett.6c00295 (PMC13492389; doi:10.1021/acsmacrolett.6c00295)
Supplement: Supplementary file 1 [file mz6c00295_si_001.pdf]

Supporting Information for:

Stiffness Tuning and Ductility Enhancement of Semicrystalline Polymers by Incorporating Miscible Noncrystallizable Blocks

*Katherine M. Gunter, Richard A. Register**

Department of Chemical and Biological Engineering, Princeton University, NJ 08544

Materials

Norbornene (Alfa Aesar, 99% purity), norbornyl norbornene, and hexylnorbornene (both provided by Promerus LLC) were purified on a Schlenk line by repeated freeze-pump-thaw cycles while stirring over sodium metal. Once the pressure gauge did not register a large deflection (roughly 10 freeze-pump-thaw cycles), the monomer was vacuum transferred through a heated short-path tube into a receiving flask, then brought into an MBraun UNIlab glovebox for polymerization. Trimethylphosphine (PMe_3, Sigma-Aldrich, 97% purity) was purified analogously without the short-path tube. 2,6-diisopropylphenylimidoneophylidenemolybdenum(VI) bis(*t*-butoxide) (used as received from Strem Chemicals), a Schrock-type metathesis catalyst, was used to initiate all polymerizations. Toluene (ThermoFisher Scientific) was purified by passing through an MBraun solvent purifier, before piping directly into the glovebox where it was used as the solvent for all polymerizations. $Ru(CO)ClH(PPh_3)_3$, denoted hRu1 below (used as received from Alfa Aesar), was employed to saturate all olefinic double bonds with H_2 (99.999% purity, Airgas) in xylene (ThermoFisher). ~20 μm thick (0.0007/0.0009 in thickness) scratch-free ruby mica was used as received from Asheville-Schoonmaker Mica Co.

Synthesis of Diblock Copolymers

All ring-opening metathesis polymerizations (ROMP) were done within the glovebox under an N_2 atmosphere, with O_2 and H_2O levels kept below 0.1 ppm. The Schrock-type initiator was dissolved in a small amount of toluene (~10 mL) before being added to a round bottom flask, where additional toluene and PMe_3 (10 eq. PMe_3 per 1 eq. initiator) were added and stirred. The PMe_3 reversibly binds to the living chain end, slowing propagation.[1] The monomer constituting

the amorphous block (either norbornyl norbornene or hexylnorbornene) was added at a concentration of 2 wt%, initiating polymerization. After 1 hour, a 1 mL aliquot was taken, and norbornene monomer dissolved in toluene (concentration of 4 wt%) was charged. The reaction was left for an additional hour to reach full conversion, before terminating with excess benzaldehyde (used as received from Sigma-Aldrich, 100 eq. per 1 eq. initiator). The polymer solution was removed from the glovebox and precipitated into methanol (ThermoFisher). Polymers were redissolved in toluene with ~0.5 wt% butylated hydroxytoluene (BHT) and precipitated again to remove terminated catalyst and other impurities. Polymers were finally dried overnight at room temperature in a vacuum oven.

Molecular Characterization Methods

^{1}H NMR spectroscopy was performed on all block copolymers using a Bruker Avance III 500 MHz UltraShield with $CDCl_3$ as the solvent. Spectra were analyzed using the MestReNova software package to confirm both sample purity and composition. Calculating the composition of the as-synthesized (unhydrogenated) PN-PHxN diblock copolymers was straightforward, as protons attached to the terminal carbon on the hexyl chain appear as a clear peak in the ^{1}H NMR spectrum at 0.87 ppm. **Figure S1** shows the ^{1}H NMR spectrum of a PHxN homopolymer, with the relevant peaks marked.

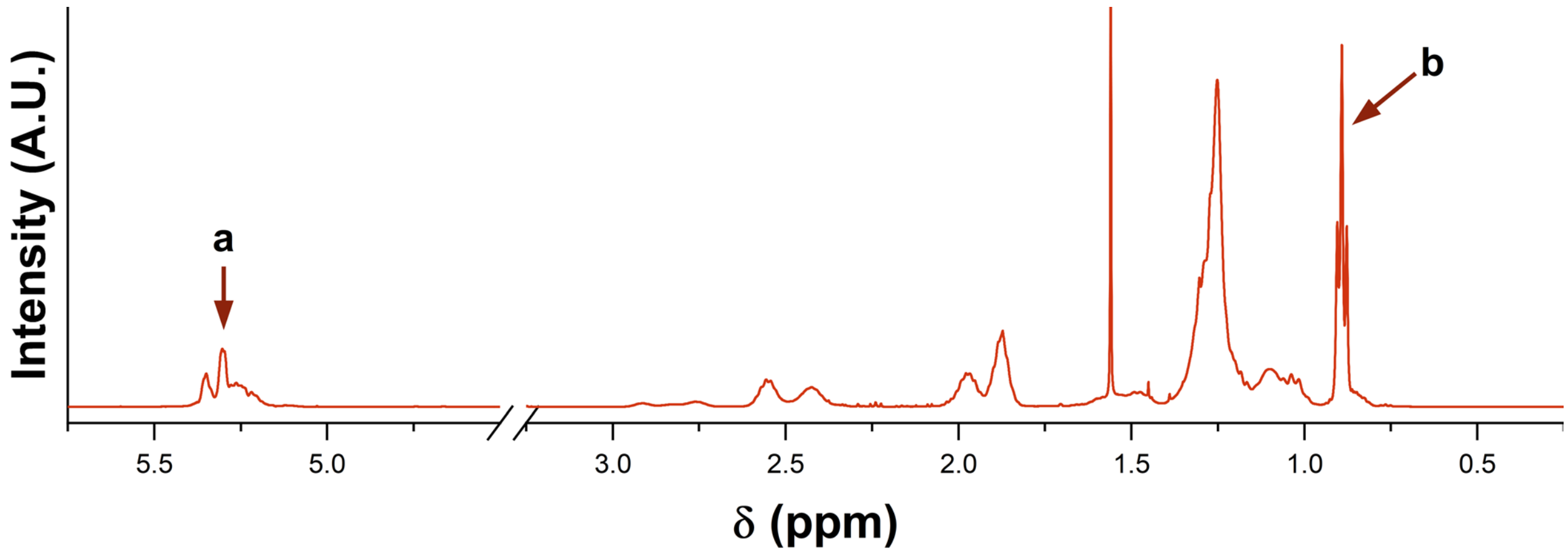

Figure S1. ^{1}H NMR spectrum of a PHxN homopolymer. Peak *a* represents the olefinic protons, and peak *b* represents protons bound to the methyl carbon on the hexyl chain. Areas underneath peaks *b* and *a* form a 3:2 ratio.

For the PN-PNbN diblocks, this determination was more complicated. The ^{1}H NMR spectrum of a PNbN homopolymer (M_n = 85.4 kg/mol) synthesized using the same Schrock-initiated ROMP protocol is shown in **Figure S2**. The peak highlighted at 0.62 ppm was calculated to form an approximately 3:10 ratio with the olefinic peaks at 5.2-5.5 ppm. While the precise identity of this 0.62 ppm peak is unknown, no such peak appears in the ^{1}H NMR spectrum of either the PN or PHxN homopolymer. We attribute this peak to protons within a particular stereoisomer of norbornyl norbornene.

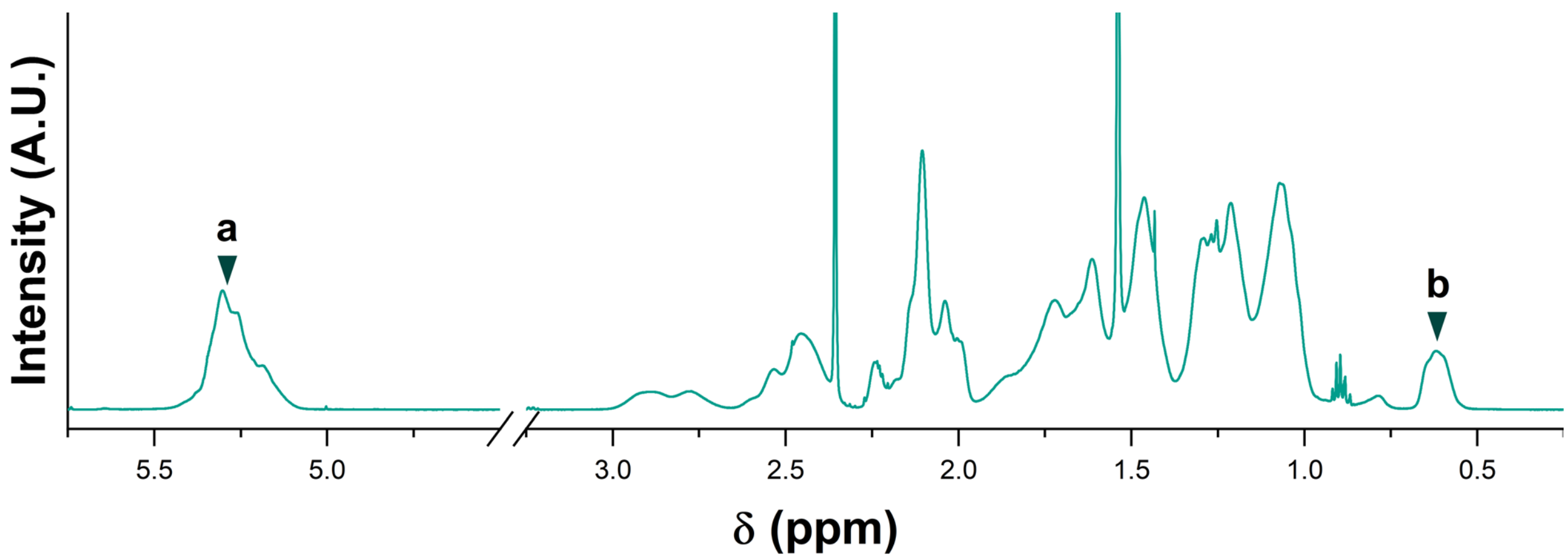

Figure S2. ^{1}H NMR spectrum of a PNbN homopolymer. Peak *a* represents the olefinic protons, and peak *b* represents protons within a specific stereoisomer of the norbornyl group. Areas underneath peaks *b* and *a* form a roughly 3:10 ratio.

Molecular weight distributions were obtained utilizing a multidetector gel permeation chromatography (GPC) system with tetrahydrofuran (THF, HPLC grade, used as received from either ThermoFisher or Sigma-Aldrich) as the mobile phase, conveyed by a Waters 515 pump at 1 mL/min through two 30 cm Agilent PLgel Mixed-C columns and into a miniDAWN TREOS three-angle light scattering detector (operating at room temperature, 658 nm), followed by a Wyatt Optilab T-rEX differential refractive index (dRI) detector (operating at 25 °C, 658 nm). The true weight-average molecular weights (M_w) of all polymers were extracted from the light scattering data using a weight-averaged dn/dc value[2] (dn/dc_{PN} = 0.1549 mL/g,[3] dn/dc_{PHxN} = 0.1110 mL/g,[4] dn/dc_{PNbN} = 0.1534 mL/g). Dispersities (Đ) were calculated from the dRI elution times calibrated with narrow-distribution polystyrene standards, which also yields the polystyrene-equivalent molecular weight ($M_{w,\mathrm{PS}}$). True molecular weight distributions were obtained by dividing the dRI trace by $M_{w,PS}/M_w$. Molecular weight distributions for the diblock copolymers are shown below in **Figure S3**.

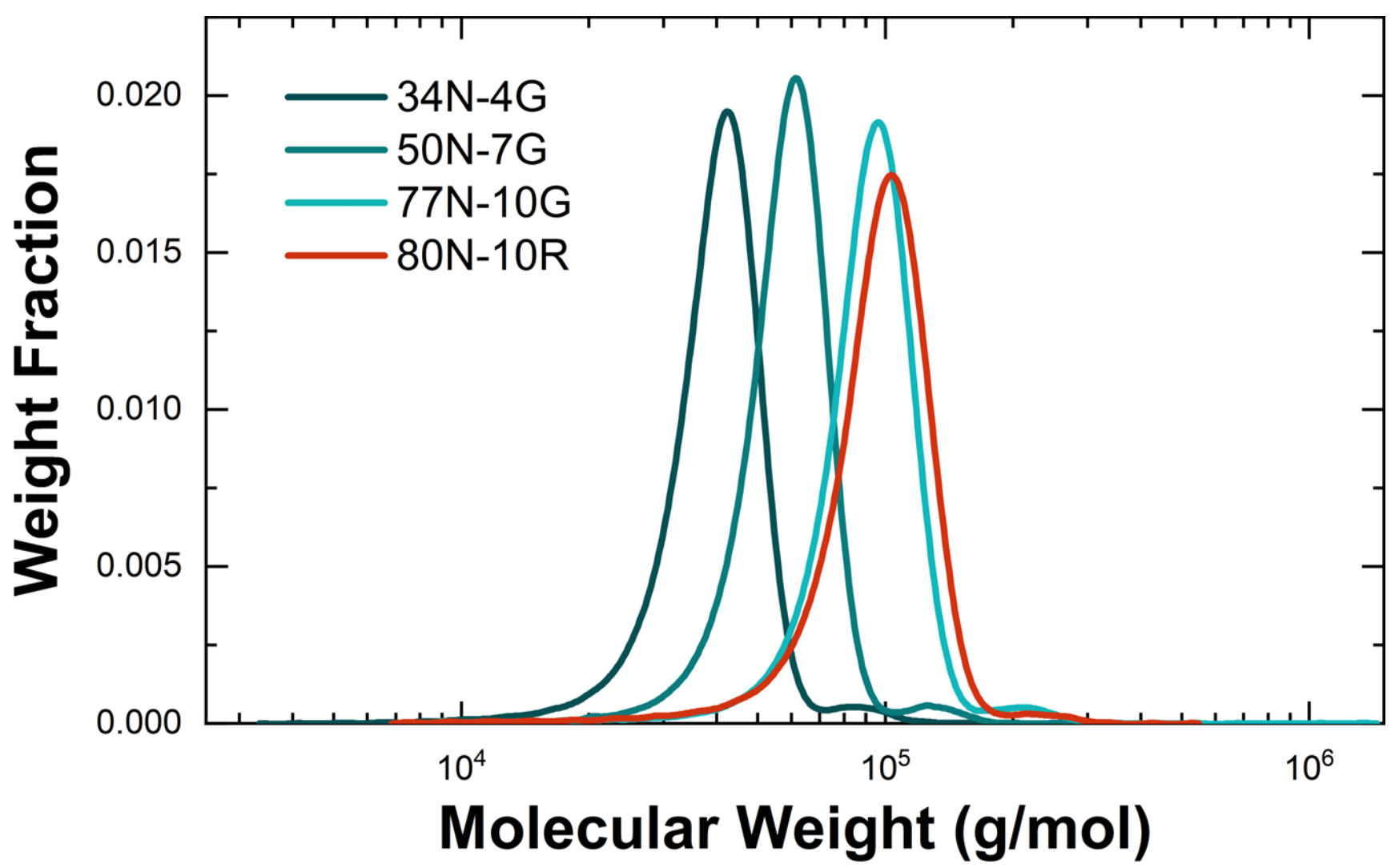

Figure S3. Molecular weight distributions of unsaturated PN-G and PN-R diblock copolymers. Note that the polymer codes represent the molecular weights of each block in the saturated material.

Hydrogenation

Catalytic hydrogenation was carried out on all block copolymers dissolved in xylene with a maximum polymer concentration of 1 wt% and a catalyst to polymer mass ratio of 6:1000. Polymers and hRu1 were dissolved overnight with ~0.5 wt% BHT before being transferred to a 2 L Parr stainless steel reactor. The reactor was flushed with N_2, pressure tested for leaks, charged with 600 psig of H_2, then heated to 165 °C with the reactor contents continuously stirred for 24 hours. After the allotted reaction time, the mixture was cooled down to room temperature before adding 500 mL of methanol and collecting the polymer precipitate by vacuum filtration. The polymer was then re-dissolved in hot toluene (~200 mL) with ~0.5 wt% BHT and precipitated into methanol to further remove any remaining hRu1. The precipitate was again recovered *via* vacuum filtration, and the hydrogenated product was dried in a vacuum oven at room temperature for 48 hours. To confirm sufficient hydrogenation of the olefinic carbons, ^{1}H NMR spectra of all

polymers dissolved in *d*-toluene were acquired using a Bruker Avance III HD 500 MHz multinuclear NMR instrument operating at 90 °C.

Figure S4 shows the ^{1}H NMR spectra of as-polymerized (unhydrogenated) and hydrogenated 77N-10G and 80N-10R. To calculate fractional hydrogenation of double bonds (f_{HYD}), **Equation (S1)** was used, where β represents the ratio of areas underneath the olefinic peaks and aliphatic peaks, and n_x represents the mole fraction of a particular block.

$$f_{HYD} = \frac{2\beta - 8n_N - 20n_{HxN} - 18n_{NbN}}{4 + 2\beta} \quad \textbf{(S1)}$$

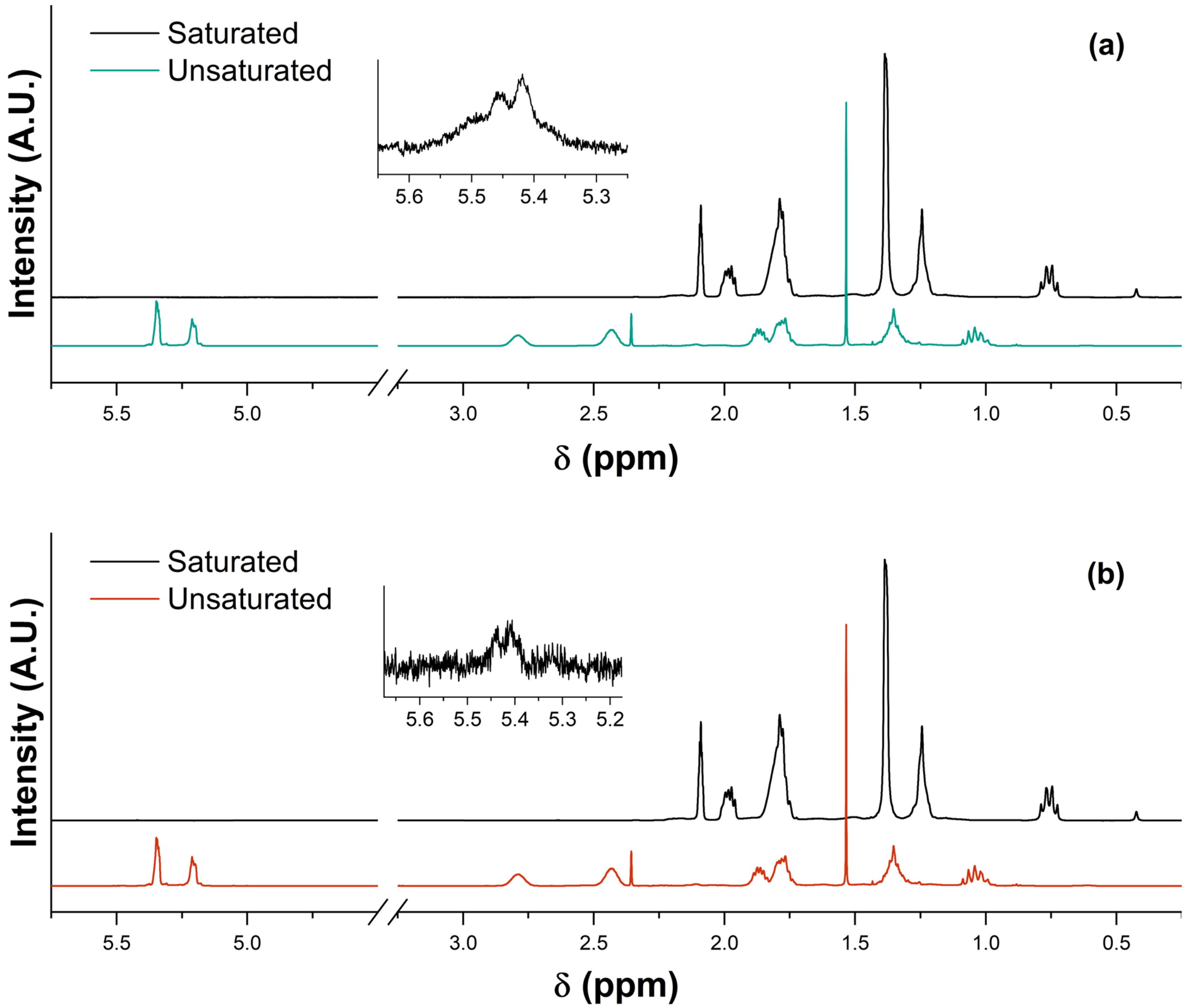

Figure S4. ^{1}H NMR spectra of (a) 77N-10G and (b) 80N-10R, as-polymerized (unsaturated) and hydrogenated (saturated). Note that the spectra for the unsaturated and saturated polymers were acquired on different instruments, in different solvents. Insets show olefinic region in saturated specimens.

Table S1 shows f_{HYD} values of all diblock copolymers. While using hRu1 with these reaction conditions leads to complete hydrogenation of PN,[5,6] complete hydrogenation of either the PNbN or PHxN block was not achieved, presumably due to steric effects. To confirm that any residual double bonds are thermally stable at 160 °C, the PNbN homopolymer whose ^{1}H NMR

spectrum is shown in **Figure S2** was hydrogenated with hRu1 for 48 hours, then repeatedly melt-pressed at 160 °C. After cooling, GPC was performed on a small section of film taken after each pressing. **Figure S5** shows the molecular weight distributions derived from the resulting dRI traces, where no significant thermal degradation was observed.

Table S1. Molecular characteristics of melt-miscible N-G and N-R diblocks.

Polymer	$M_{n,saturated}$ (kg/mol)	Đ	w_{hPNbN}[a]	w_{hPHxN}[a]	f_{HYD} (%)
34N-4G	38.3	1.09	0.117	0	98.6
50N-7G	57.2	1.07	0.121	0	98.5
77N-10G	86.6	1.12	0.110	0	99.0
80N-10R	90.0	1.13	0	0.112	99.9

a. Weight fraction of the indicated amorphous block in the saturated specimen

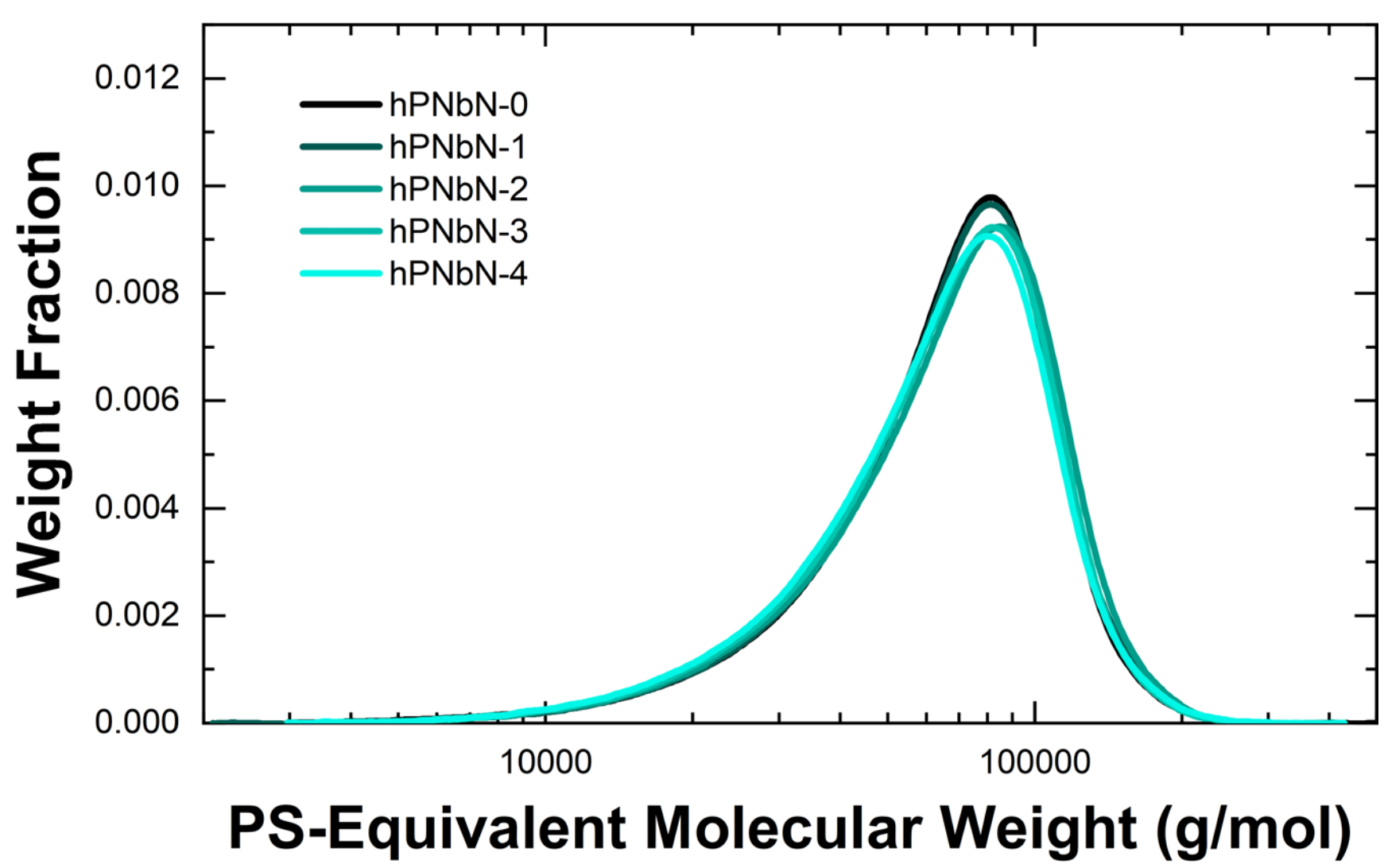

Figure S5. PS-equivalent molecular weight distributions of hPNbN (G) homopolymer ($M_{n,unsaturated}$ = 85.4 kg/mol) following repeated melt-pressings at 160 °C. The number in the sample code indicates the number of times the sample was melt-pressed.

Film Preparation and Crystallization

Polymer films 0.2 – 0.4 mm thick were prepared by melt-pressing each between poly(ethylene terephthalate), PET, sheets using a PHI press equipped with heated platens set to 160 °C. The molten polymer film was held at 160 °C for 5 minutes to erase any previous thermal history. The PET-polymer film-PET sandwich was then either immediately submerged into tap water (quenching, ~1000 °C/min) or ambiently cooled on the PHI press overnight after shutting off the heating plates (slow-cooling, < 1 °C/min).

Small Angle X-ray Scattering

Room-temperature, one-dimensional SAXS (RT-SAXS) patterns were obtained on all films by cutting out and stacking rectangular samples and affixing them within an Anton-Paar compact Kratky camera. CuKα X-rays (λ = 0.15418 nm) were produced with a PANalytical PW3830 generator fitted with a long-fine-focus Cu tube, and scattered X-rays were measured on an MBraun OED-50 M position-sensitive detector. RT-SAXS patterns were acquired for 1 hour. All 1D SAXS data were then converted to absolute intensity units using a polyethylene calibrant[7] and desmeared using an iterative method developed by Lake.[8] SAXS data are presented in terms of the magnitude of the momentum transfer vector $q = (4\pi/\lambda)\sin(\theta)$, where θ is half the scattering angle. RT-SAXS patterns for all diblock copolymer films are shown in **Figure S6**. The *d*-spacing was calculated using Bragg's Law, $d = 2\pi/q^*$, where q^* is the position of the primary SAXS peak in a plot of q^2I vs. q.

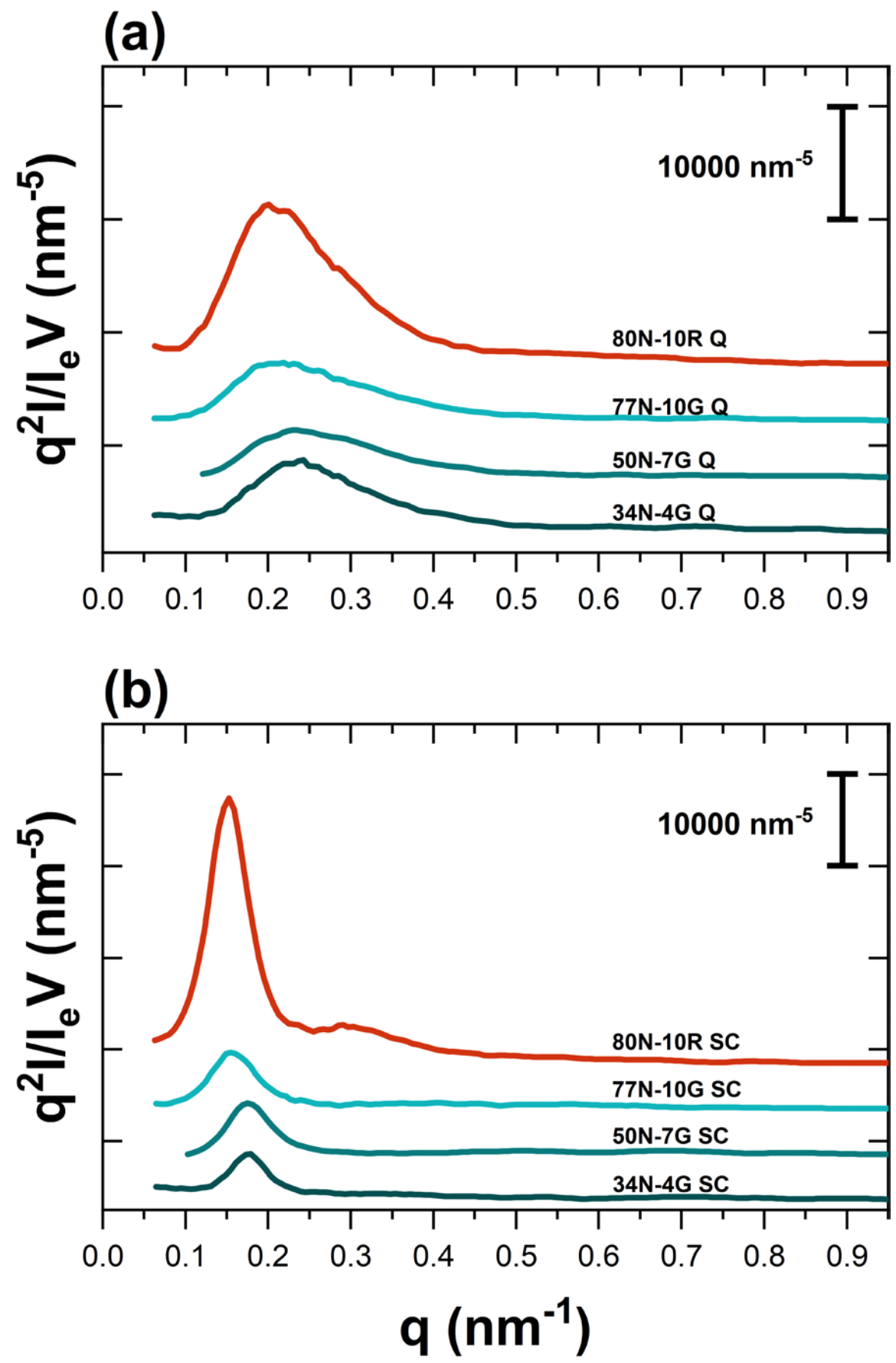

Figure S6. Room-temperature SAXS patterns of N-G and N-R specimens (a) quenched and (b) slow-cooled from the melt. Patterns are vertically offset for visual clarity.

High-temperature SAXS (HT-SAXS) patterns were taken using the same compact Kratky camera, but specimens were instead affixed into a custom-built hot stage controlled with an Omega 1/16 DIN Universal Temperature and Process Controller. Samples were melt-pressed into copper molds between a pair of mica sheets, each ~20 μm thick, using the PHI press described above. The hot-stage temperature was calibrated with HDPE (Dow), LDPE (Dow), and *i*PP (Himont), with the calibration curve shown in **Figure S7**. For this calibration, the melting temperature (midpoint of transition) of each polymer measured by differential scanning calorimetry (DSC, see following section) was compared to the melting temperature determined by SAXS analysis. This difference

is the y-coordinate of our calibration curve. To ensure identical thermal histories between the DSC and SAXS samples, the aluminum DSC pan was placed on the melt press while copper molds of each polymer were prepared. Both the DSC and SAXS specimens were slow-cooled overnight. For the ambient temperature measurement, the temperature on a glass alcohol thermometer was compared to the temperature reading on the thermocouple.

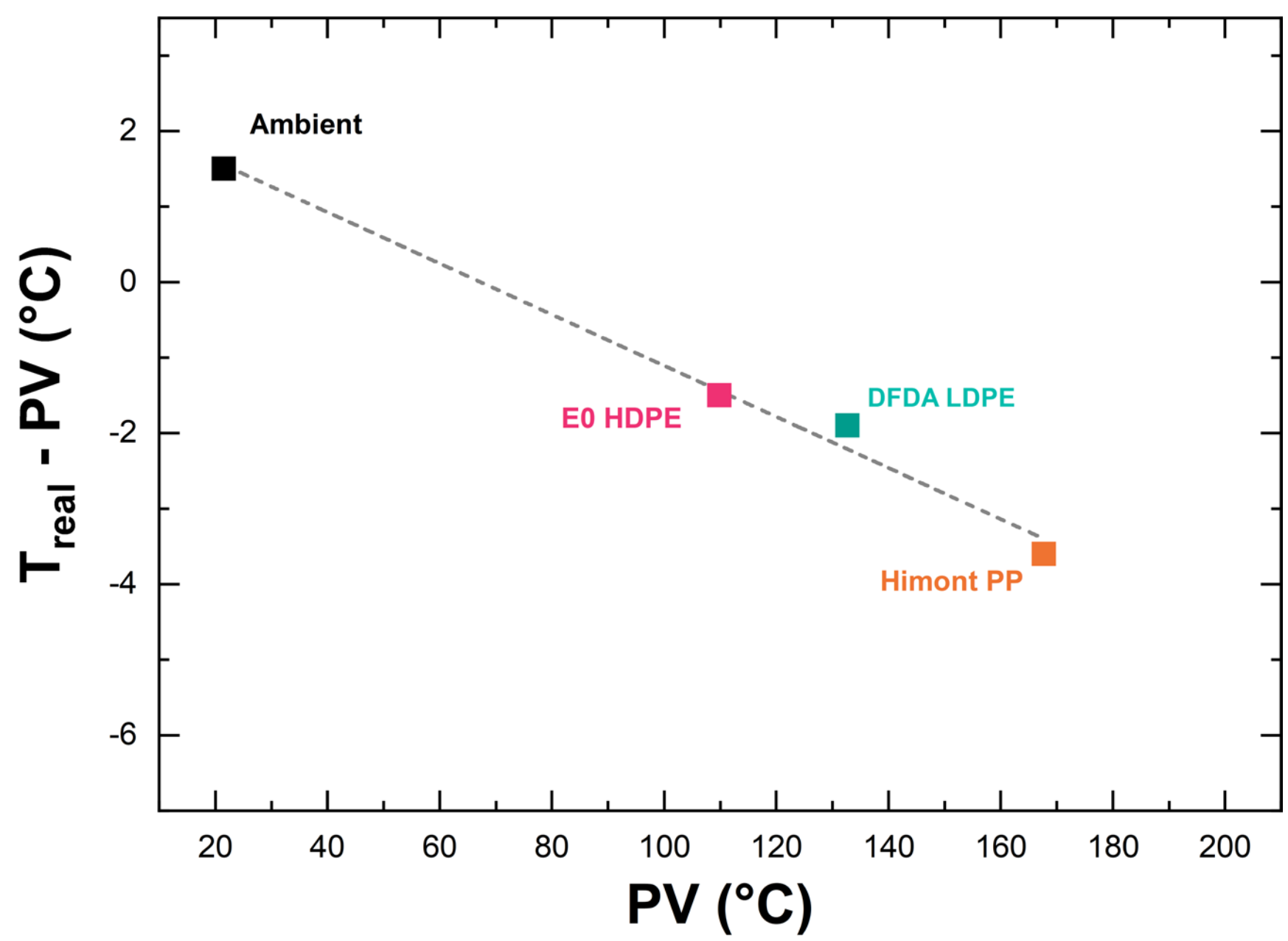

Figure S7. Temperature calibration curve of the custom Kratky hot stage, plotted as the difference between the actual sample temperature (T_{real}) and the thermocouple reading (PV) against PV.

Thermal Analysis

First-heat melting endotherms were acquired with a PerkinElmer DSC7, calibrated with indium and tin. A small disc of material (~2 mg) was stamped from the melt-pressed polymer film and sealed in an aluminum pan. The sample was then heated from 25 °C to 160 °C at a ramp rate of 10 °C/min. **Figure S8** shows the first-heat melting endotherms of the diblock copolymers.

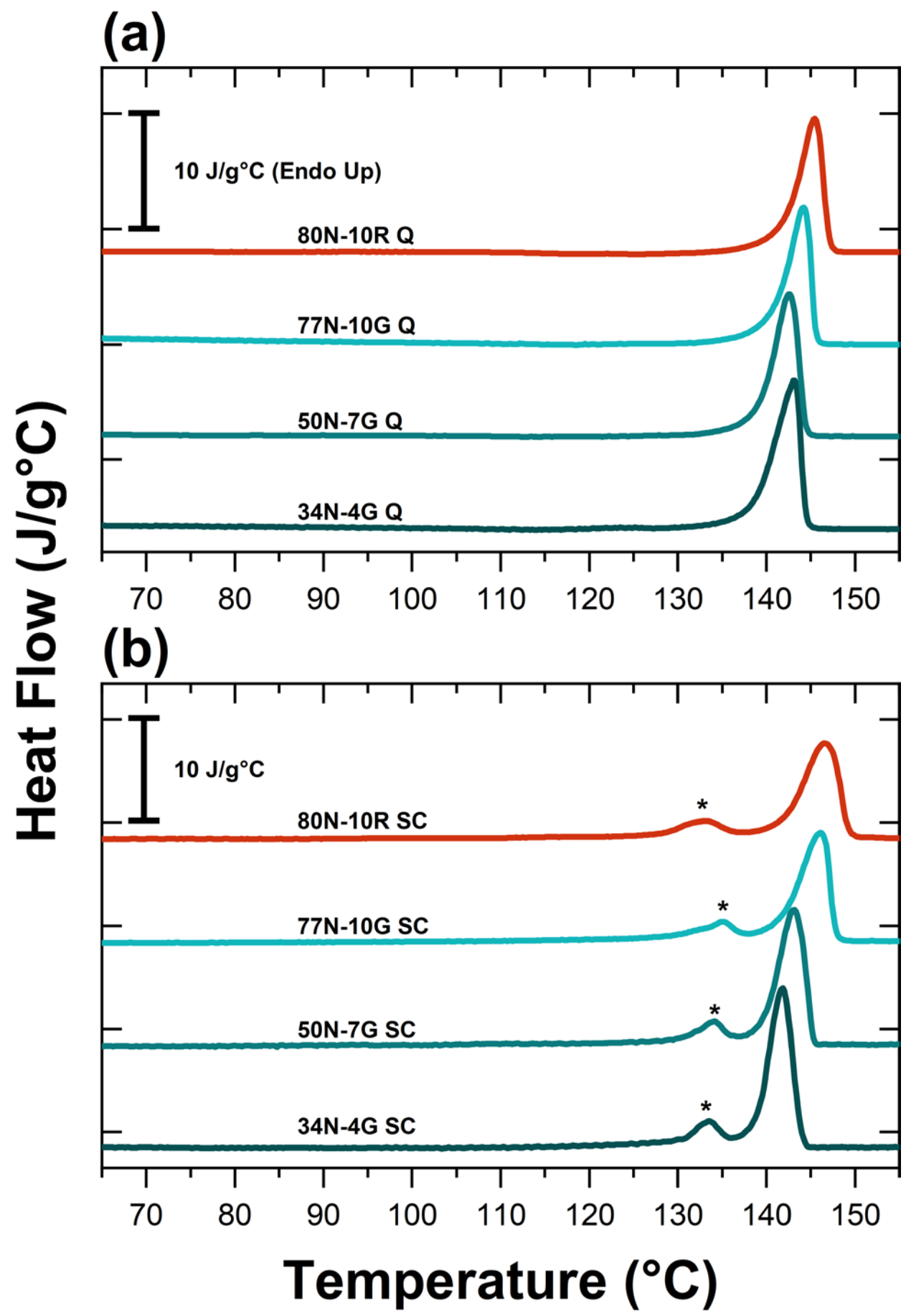

Figure S8. First-heat melting endotherms of (a) quenched and (b) slow-cooled diblock copolymers. Subsidiary peaks in (b), marked by asterisks, are believed to be the crystal-crystal polymorphic transition (T_{cc}) within the N crystals[9,10]

Melting enthalpies were calculated by first subtracting the thermogram of an empty cell from the sample thermogram, then integrating the area underneath the endothermic peak. Weight fraction crystallinity (w_c) was calculated by dividing the melting enthalpy by 96 J/g, the melting enthalpy of the pure, unepimerized hPN crystal.[6] Volume fraction crystallinity (φ_c) was calculated from w_c using **Equations S2-S3**:

$$\varphi_c = \frac{\frac{w_c}{\rho_c}}{\frac{w_c}{\rho_c} + \frac{w_a}{\rho_a}} \tag{S2}$$

$$\frac{1}{\rho_a} = \left(1 - \frac{w_{hPaN}}{1 - w_c}\right)\frac{1}{\rho_{hPN,a}} + \frac{w_{hPxN}}{(1 - w_c)}\frac{1}{\rho_{hPaN}} \tag{S3}$$

where ρ_c is the room-temperature density of crystalline hPN (1.0191 g/cm^3),[9] ρ_a is the amorphous phase density, $\rho_{hPN,a}$ is the density of amorphous hPN at 23 °C (0.9585 g/cm^3),[11] and ρ_{hPaN} is the density of the homopolymer corresponding to the amorphous block at 23 °C (1.005 g/cm^3 and 0.909 g/cm^3 for hPNbN and hPHxN, respectively).[4] **Table S2** lists relevant thermal properties and morphological dimensions for these diblock copolymers. Crystal thickness (L_c) was calculated by assuming an ideal two-phase model (i.e., $L_c = d \cdot \varphi_c$).

Table S2. Thermal properties and morphological dimensions of diblock copolymers.

Polymer	Peak T_m (°C)	Peak T_{cc} (°C)	ΔH_m (J/g)	w_c	φ_c	d (nm)	L_c (nm)
34N-4G Q	143	*a*	51.6	0.537	0.525	26.4	13.9
50N-7G Q	142	*a*	48.0	0.500	0.487	26.4	12.9
77N-10G Q	144	*a*	44.6	0.464	0.452	29.3	13.2
80N-10R Q	145	*a*	43.9	0.457	0.439	30.0	13.2
34N-4G SC	142	134	65.9	0.687	0.677	35.8	24.2
50N-7G SC	143	134	59.8	0.623	0.612	35.6	21.8
77N-10G SC	146	135	59.2	0.617	0.605	40.1	24.3
80N-10R SC	147	133	60.6	0.632	0.613	41.2	25.3

a. No T_{cc} observed.

Mechanical Analysis

Stress-strain curves for all films were acquired with an Instron 5865 equipped with a 1 kN load cell. Three dogbones were stamped from each film using an ASTM D1708 die (2.22 cm gauge length). Each dogbone was then mounted in a uniaxial tensile test crosshead and strained at a rate of 2.54 cm/min until failure. A total of three replicates for each film were tested.

Strain-hardening moduli ($\langle G_p \rangle$) for all materials were calculated using the method outlined by Kurelec et al.[12] First, engineering stress was converted to true stress using **Equation S4** and the stretch ratio (λ, equal to 1 + ε), approximating Poisson's ratio as ½. The slope over the strain-hardening region was averaged with **Equation S5**.

$$\sigma_{true} = \lambda \sigma_{eng} \quad \textbf{(S4)}$$

$$\langle G_p \rangle = \frac{1}{N} \sum_{i=1}^{N} \frac{\sigma_{true,i+1} - \sigma_{true,i}}{\lambda_{i+1} - \lambda_i} \quad \textbf{(S5)}$$

Diblock Copolymers with Microphase-Separated Melts

Two additional, higher-molecular-weight N-G diblock copolymers were synthesized, the characteristics of which are listed in **Table S3**. HT-SAXS revealed that both exhibited structured melts prior to crystallization (**Figure S9**). These features are most evident in 149N-18G, where the SAXS pattern adopts a shape suggesting the formation of spherical microdomains with a liquid-like packing.[13] The observation of a clear SAXS peak in 149N-18G confirms that sufficient electron density contrast exists between hPN and hPNbN at 160 °C for SAXS to readily reveal microphase-separation in such N-G diblocks, when present. For 106N-14G, such features appear only after cooling the specimen down to 145 °C, indicating a weakly phase-separated melt.

Table S3. Molecular characteristics of diblock copolymers with microphase-separated melts.

Polymer	$M_{n,saturated}$ (kg/mol)	Đ	w_{hPNbN}[a]	f_{HYD} (%)
106N-14G	120.3	1.21	0.121	98.8
149N-18G	166.7	1.15	0.107	99.0

a. Weight fraction of the amorphous block in the saturated specimen

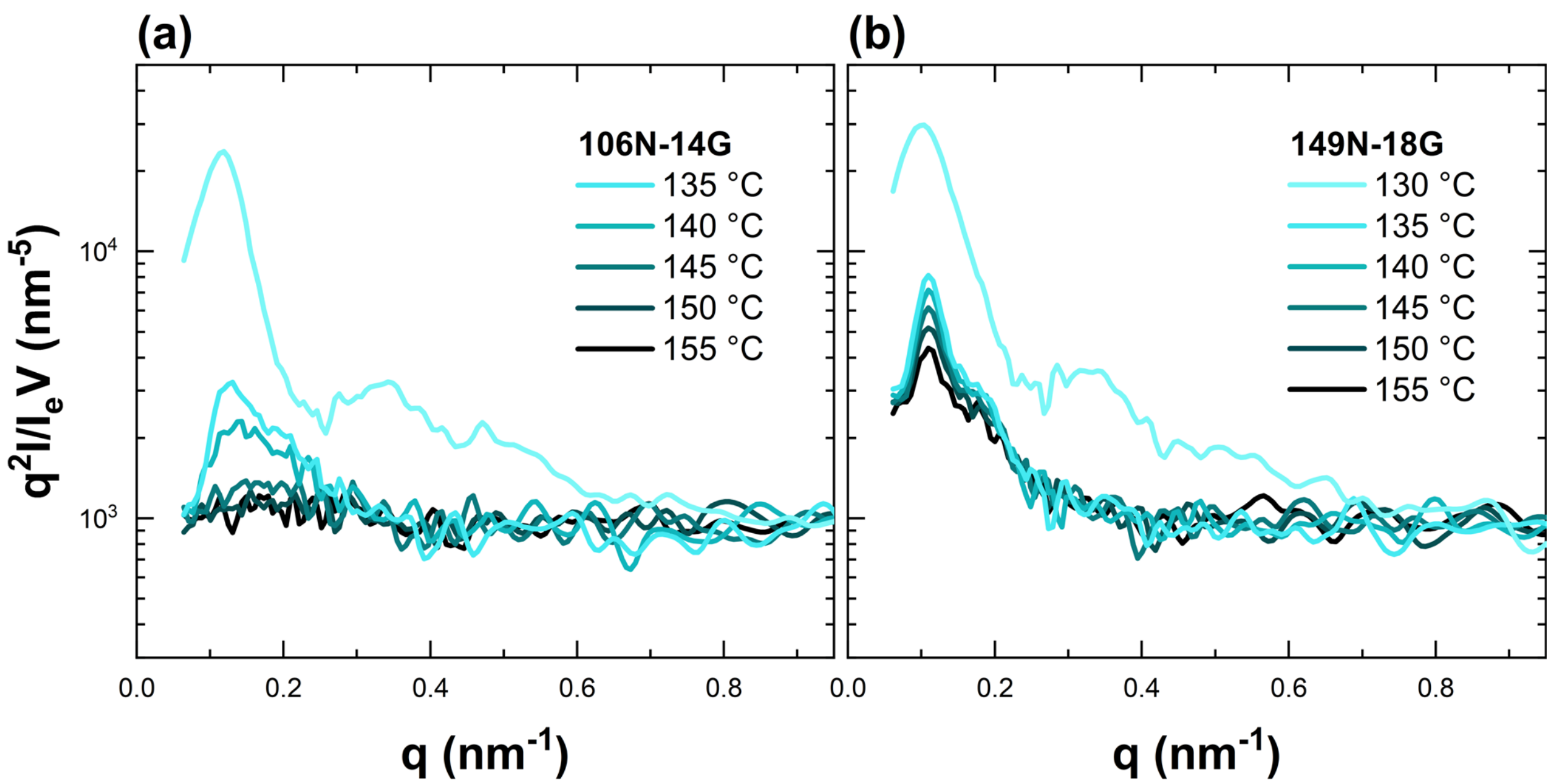

Figure S9. HT-SAXS patterns of (a) 106N-14G and (b) 149N-18G cooled *in situ*.

No N-R diblocks of higher M_n than 80N-10R were synthesized to probe the onset of melt microphase separation in the N-R system. However, sufficient electron density contrast between molten N and R (hPN and hPHxN) should be present to observe microphase separation, were it present in 80N-10R (which has a featureless SAXS pattern, Figure 1(b)). The density[11] of molten hPN at 160 °C is 0.8786 g/cm^3; with the measured density of hPHxN at 23 °C (0.909 g/cm^3) and its measured thermal expansion coefficient[4] $\alpha = 5.70 \times 10^{-4}$ °C^{-1}, its density at 160 °C is calculated as 0.841 g/cm^3. From these values, the electron density difference between hPN and hPHxN is

calculated to be 11 e^-/nm^3 at 160 °C. This value of 11 e^-/nm^3 is identical to that calculated for a lamellar diblock of different chemistry (hI-(S*r*hI)$_{24}$-97), where SAXS measurements on the same apparatus revealed a clear peak in the microphase-separated state, as well as a clear order-disorder transition upon heating.[14]

Mechanical testing was performed on quenched and slow-cooled films of the two higher-M_n N-G diblocks, following crystallization from their microphase-separated melts. Stress-strain curves are shown below in **Figure S10**, with values for mechanical properties summarized in **Table S4**. When quenched (**Figure S10a**), both 106N-14G and 149N-18G exhibit substantially reduced ductility compared to 77N-10G, as evidenced by the reduced strain at break (ε_b) and lack of pronounced strain-hardening. By contrast, 106N-14G SC and 149N-18G SC both fail in a ductile fashion and demonstrate strain-hardening behavior (**Figure S10b**). Both σ_y and E drop to below the values for the N homopolymer of an identical thermal history, which can be partially attributed to a decrease in crystallinity, but also reflects the immiscibility of the N and G blocks in these higher-M_n materials.

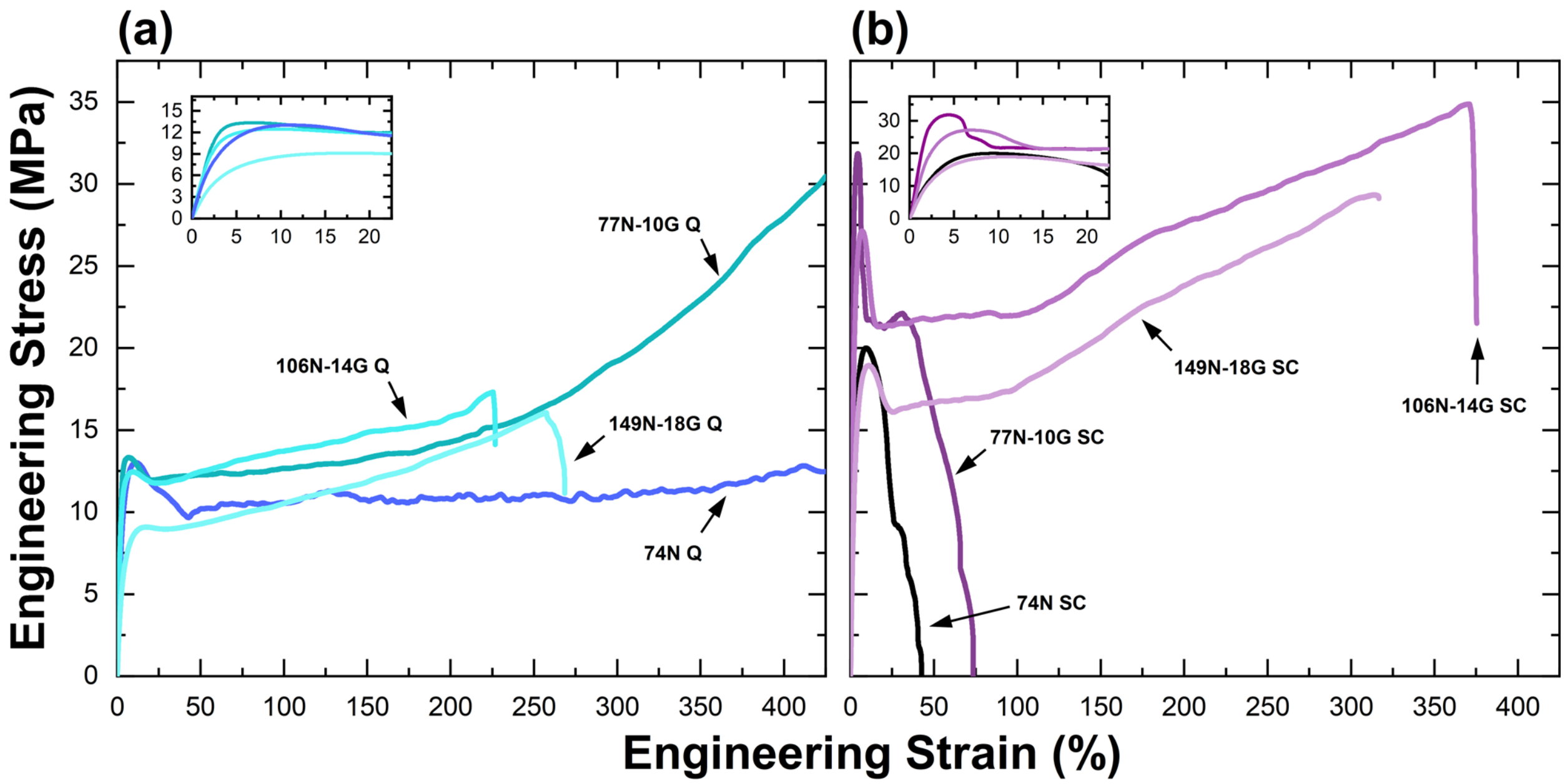

Figure S10. Stress-strain curves for high-M_n N-G diblock copolymers (a) quenched and (b) slow-cooled from the melt, in comparison to 77N-10G and 74N. Insets show the low-strain region.

Table S4. Mechanical properties of N-G diblock copolymers with microphase-separated melts.[a]

Polymer	ε_b (%)	σ_y (MPa)	E (MPa)[b]	$\langle G_p \rangle$ (MPa)
106N-14G Q	220 ± 45	12.8 ± 0.3	520 ± 20	22 ± 5
149N-18G Q	270 ± 18	9.0 ± 0.5	250 ± 4	26 ± 5
106N-14G SC	360 ± 25	27.5 ± 0.3	1230 ± 105	42 ± 4
149N-18G SC	310 ± 79	19.1 ± 0.2	640 ± 34	50 ± 5

a. Average ± one standard deviation of three replicates
b. Young's modulus, defined as the slope of the stress-strain curve during initial (<1%) deformation

When compared to the lower-M_n N-G diblocks, $\langle G_p \rangle$ is observed to decrease when the specimen is crystallized from a structured melt. Looking first at the quenched specimens, increasing M_n from 87 to 120 kg/mol results in a 75% drop in $\langle G_p \rangle$ (87 MPa to 22 MPa). The G blocks, which were confined to spherical microdomains at the point of crystallization,[13] no longer

beneficially reduce tie molecule loss during drawing. For the slow-cooled specimens, ⟨G_p⟩ is similar to that for a high-molecular weight, slow-cooled N homopolymer (42 MPa).[6]

Tie Molecule Calculation

Using the solid-state dimensions and molecular weight distributions of all materials, it is possible to calculate the tie molecule content (P_{avg}, fraction of chains forming tie molecules) in the polymer films, allowing for further assessment of how the brittle-ductile transition for these materials compares to that for N homopolymers. P_{avg} was calculated by first calculating the probability of tie molecule formation for each chain length in the sample (**Equations S6-S8**), then averaging these values over the full molecular weight distribution (**Equation S9**), an approach first developed by Huang and Brown:[15,16]

$$P = \frac{1}{3}\frac{\int_{L_{crit}}^{\infty} r^2\, exp(-B^2r^2)\, dr}{\int_0^{\infty} r^2\, exp(-B^2r^2)\, dr} \tag{S6}$$

$$B^2 = \frac{3}{2R_0^2} \tag{S7}$$

$$R_0^2 = kM \tag{S8}$$

$$P_{avg} = \frac{\int_0^{\infty} nP(M)\, dM}{\int_0^{\infty} n\, dM} \tag{S9}$$

where R_0^2 is the mean squared end-to-end distance of the polymer chain (taken to be identical in the melt and solid states[17,18]), L_{crit} is the minimum length a chain needs to span to form a tie molecule (set here to $L_c + d$),[16] and k is the chain stiffness factor (equal to 1.014 Å^2 mol/g for molten hPN[19]). Because only the N block can crystallize, and the amorphous block is positioned at the chain end, the portion of a polymer molecule which spans L_{crit} is limited to the N block.

Figure S11 shows this concept schematically. To correct for this, each value of M in the complete molecular weight distribution is multiplied by the mass fraction of N in the diblock.

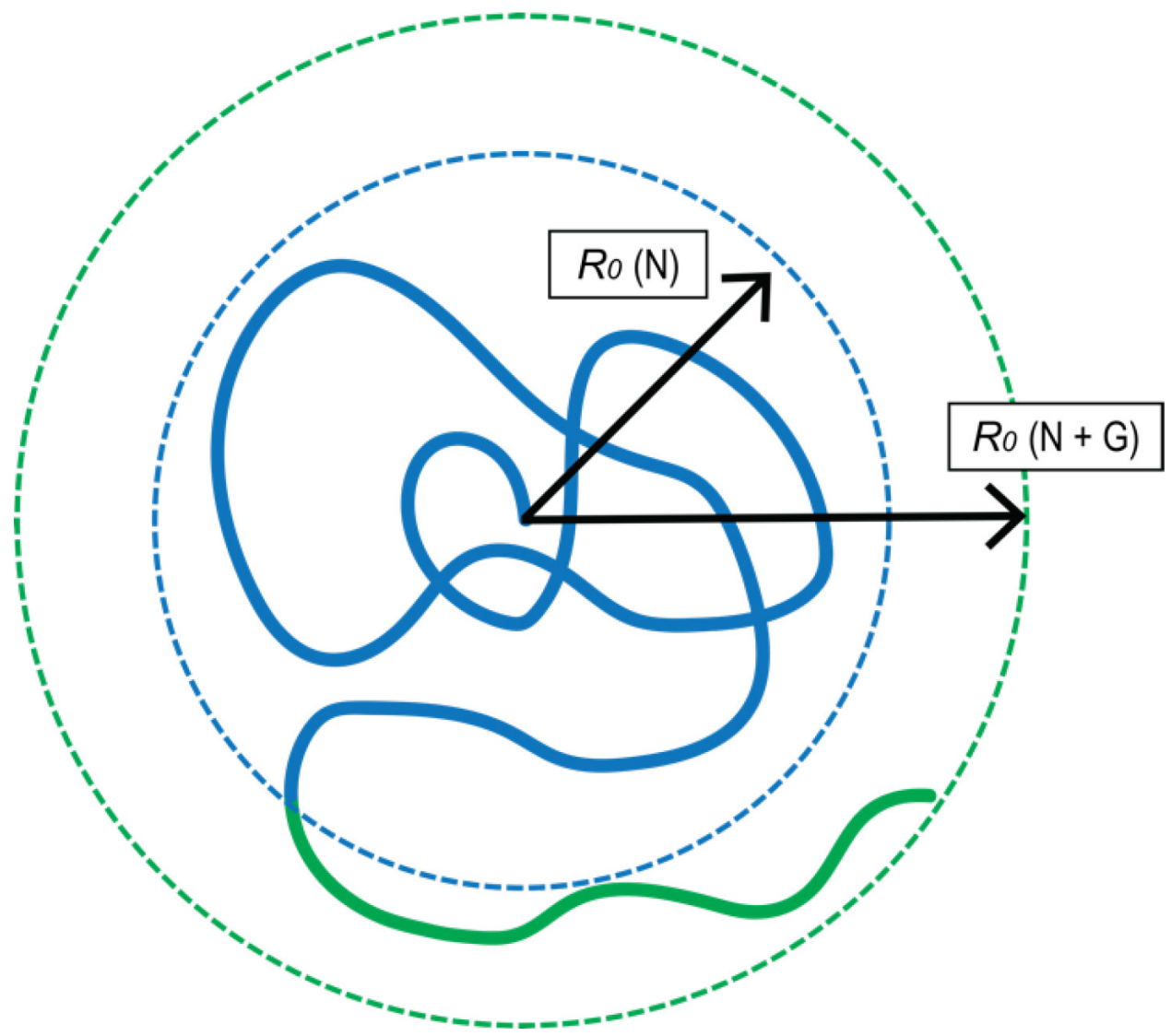

Figure S11. Schematic of an N-G diblock copolymer in the melt, highlighting the difference in R_0 between the N block in isolation and the full molecule.

References

(1) Og, J. H. Kinetic Constants and Reactivity Ratios for Norbornene-Hexylnorbornene Statistical Copolymerization. M.S.E. Thesis, Princeton University, 2021.

(2) Bushuk, W.; Benoît, H. Light-Scattering Studies of Copolymers: I. Effect of Heterogeneity of Chain Composition on the Molecular Weight. *Can. J. Chem.* **1958**, *36*, 1616–1626.

(3) Burns, A. B.; Register, R. A. Large, Reversible, and Coherent Domain Spacing Dilation Driven by Crystallization under Soft Lamellar Confinement. *Macromolecules* **2017**, *50*, 8106–8116.

(4) Mulhearn, W. D.; Register, R. A. Melt Miscibility in Diblock Copolymers Containing Polyethylene and Substituted Hydrogenated Polynorbornenes. *Macromolecules* **2017**, *50*, 5830–5838.

(5) Klein, J. P. Structure and Property Control and Crystallite Influence on Segmental Dynamics in Hydrogenated Polynorbornene. Ph.D. Thesis, Princeton University, 2022.

(6) Gunter, K. M.; Register, R. A. Role of Crystal Thickness on the Critical Tie Molecule Fraction in Semicrystalline Polymers. *Macromolecules* **2025**, *58*, 10800–10809.

(7) Register, R. A.; Bell, T. R. Miscible Blends of Zinc-neutralized Sulfonated Polystyrene and Poly(2,6-dimethyl 1,4-phenylene Oxide). *J. Polym. Sci. B. Polym. Phys.* **1992**, *30*, 569–575.

(8) Lake, J. A. An Iterative Method of Slit-Correcting Small Angle X-Ray Data. *Acta Cryst* **1967**, *23*, 191–194.

(9) Lee, L.-B. W.; Register, R. A. Hydrogenated Ring-Opened Polynorbornene: A Highly Crystalline Atactic Polymer. *Macromolecules* **2005**, *38*, 1216–1222.

(10) Bishop, J. P.; Register, R. A. The Crystal–Crystal Transition in Hydrogenated Ring-opened Polynorbornenes: Tacticity, Crystal Thickening, and Alignment. *J. Polym. Sci. B. Polym. Phys.* **2011**, *49*, 68–79.

(11) Lee, L.-B. W. Polymer Crystalline Texture Controlled Through Film Blowing and Block Copolymerization. Ph.D. Thesis, Princeton University, 2004.

(12) Kurelec, L.; Teeuwen, M.; Schoffeleers, H.; Deblieck, R. Strain Hardening Modulus as a Measure of Environmental Stress Crack Resistance of High Density Polyethylene. *Polymer* **2005**, *46*, 6369–6379.

(13) Adams, J. L.; Quiram, D. J.; Graessley, W. W.; Register, R. A.; Marchand, G. R. Ordering Dynamics of Compositionally Asymmetric Styrene−Isoprene Block Copolymers. *Macromolecules* **1996**, *29*, 2929–2938.

(14) Beckingham, B. S.; Register, R. A. Regular Mixing Thermodynamics of Hydrogenated Styrene–Isoprene Block–Random Copolymers. *Macromolecules* **2013**, *46*, 3084–3091.

(15) Huang, Y.-L.; Brown, N. The Effect of Molecular Weight on Slow Crack Growth in Linear Polyethylene Homopolymers. *J Mater Sci* **1988**, *23*, 3648–3655.

(16) Huang, Y.; Brown, N. Dependence of Slow Crack Growth in Polyethylene on Butyl Branch Density: Morphology and Theory. *J. Polym. Sci. B. Polym. Phys.* **1991**, *29*, 129–137.

(17) Dettenmaier, M.; Fischer, E. W.; Stamm, M. Calculation of Small-Angle Neutron Scattering by Macromolecules in the Semicrystalline State. *Colloid Polymer Sci* **1980**, *258*, 343–349.

(18) Tanzer, J. D.; Bartels, C. R.; Crist, B.; Graessley, W. W. Dimensions of Polymer Chains in the Semicrystalline Solid State. *Macromolecules* **1984**, *17*, 2708–2714.

(19) Cho, S. H. Role of Tie Molecules in Ductility and Chain Deformation of Polyethylene. Ph.D. Thesis, Princeton University, 2022.
